# The Aging Lung: Exploring Multimorbidity Patterns and Their Clinical Implications: A Narrative Review

**DOI:** 10.3390/cimb47070561

**Published:** 2025-07-18

**Authors:** Ali Albarrati, Nichola S. Gale

**Affiliations:** 1 School of Healthcare Sciences, Cardiff University, University Hospital of Wales, Cardiff CF14 4XN, UK; galens@cf.ac.uk; 2 College of Applied Medical Sciences, King Saud University, Riyadh 11451, Saudi Arabia

**Keywords:** Aging, multiple chronic conditions, chronic disease, lung

## Abstract

Aging is a multifaceted biological process characterized by a progressive decline in cellular function and physiological resilience, increasing the risk of multiple chronic conditions. Chronic lung diseases frequently manifest within the aging population and are closely intertwined with systemic dysfunctions across cardiovascular, musculoskeletal, and neurological systems. In this review, we explore the biological mechanisms linking aging, multiple chronic conditions patterns, and chronic lung disease, with a particular focus on inflammaging and cellular aging. We also highlight shared pathological pathways such as oxidative stress, mitochondrial dysfunction, and the dysregulation of repair processes that underlie both natural aging and the accelerated aging seen in chronic lung disease. Additionally, we discuss the systemic impact of multiple chronic conditions on patient outcomes, including increased frailty, diminished physical capacity, cognitive impairment, and elevated mortality risk. This review advocates for a comprehensive, patient-centered approach that combines early detection, personalized pharmacological therapies targeting inflammatory and senescent pathways, and non-pharmacological interventions such as pulmonary rehabilitation, exercise, and dietary optimization. Emerging therapeutics, including senolytics and anti-inflammatory agents, present promising avenues for mitigating age-related lung decline and managing multiple chronic conditions.

## 1. Introduction

Advancing age is associated with the accumulation of failing physiological systems, manifested as multiple disorders that occur at various ages. Multimorbidity, having two or more chronic conditions in the same individual, is increasingly being recognized as a major health problem in the aging population [1,2,3,4]. Multimorbidity, comprising both physiological and psychological conditions, increases with age, with a greater than twofold increase from a mean of 1.18 morbidities per individual, with a 30% multiple chronic conditions rate, in 45–64-year-olds to a mean 2.60 morbidities per individual, with a 65% multiple chronic conditions rate, in 65–84-year-olds [2]. However, the prevalence of multiple chronic conditions in various populations is multifactorial [1,2,3,4]. A multimorbid state is associated with increased mortality, as well as reduced functional status and quality of life. Globally, the population is aging, with an estimated 2 billion individuals aged over 60 years, representing over 20% of the population by 2050. This will result in significant implications for individuals and society, with increased healthcare costs and the need to redesign patterns of care to meet the needs of individuals [1,2,3,4,5].

The term comorbidity has been widely used to describe disorders occurring in the context of an existing disease state. This approach has, until recently, hampered consideration of the possibility that many chronic disorders, such as chronic obstructive pulmonary disease (COPD), represent elements of a wider state of multiple chronic conditions, as defined above [1,2,3,4,6]. Supporting this view, chronic diseases, such cardiovascular disease (CVD) and Type 2 diabetes mellitus (T2D) appear to share common underlying mechanisms that underlie aging and the associated multiple chronic conditions [5,6]. In this narrative review, we examine the clustering of chronic conditions frequently observed in the aging population, and chronic lung diseases. We present evidence that many co-occurring diseases are not unique pathologies, but have similar biological mechanisms and interactive pathophysiological processes.

## 2. Mechanisms of Aging and Multimorbidity

Aging and chronic lung disease share similar systemic manifestations and probably share underlying mechanisms. There have been several theories of how “natural” aging occurs and the underlying mechanisms [7]. Healthy aging and life span were previously considered to be genetically determined, but the latest evidence shows genetics has a modest effect of 25–35% [8,9]. The results from Swedish and Dannish registries have shown that genetic differences account for about 25% of the variance in adult human lifespans [10,11]. Similarly, family studies have demonstrated that approximately 25% of the variation in human longevity is due to genetic factors [12]. Recently, a study by Argentieri et al. analyzed data from nearly 500,000 British participants in the UK Biobank project [9]. This study examined the influence of genetic profiles and environmental exposure to 164 factors on susceptibility to 22 major age-related chronic diseases and premature mortality. They found that environmental factors collectively were about ten times more important than genetic factors in predicting premature mortality [9].

The major factors are now believed to be environmental, such as exposure to risk factors including pollution and lifestyle factors [8,12,13]. The decline in healthy behaviors including, physical, nutritional, and socioeconomic factors results in an increased risk of comorbidities, such as obesity, hypercholesterolemia, hypertension, and diabetes mellitus [13]. Over the last century, this has led to non-communicable diseases such as COPD and CVD replacing infectious diseases as the dominant health care burden, as almost all chronic conditions are strongly related to aging [14].

**Figure 1:**
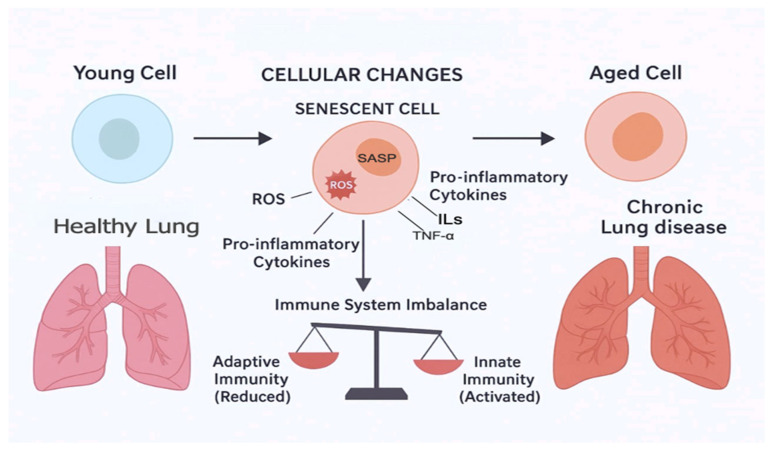
Process of inflammaging and development of chronic lung diseases.

With advanced aging, individuals are increasingly more susceptible to chronic diseases that weaken their normal physiological response to internal and external stressors (Figure 1) [15,16]. Advanced aging, whether natural or accelerated, involves changes at molecular and subcellular levels, including excess oxidant stress causing deleterious effects on DNA, proteins, and lipids [16,17,18]. Another factor that may be involved in aging is cellular aging [19]. Cellular aging, a hallmark of aging, is where cells permanently cease to divide in response to stressors such as DNA damage or oxidative stress [19,20,21]. These senescent cells secrete a variety of pro-inflammatory cytokines, chemokines (CXCs), growth factors, and proteases, collectively known as the cellular aging-associated secretory phenotype (SASP) [21]. The accumulation of senescent cells and their SASP factors contributes to tissue dysfunction and chronic inflammation, thereby playing a significant role in the development of chronic diseases [16,17]. Furthermore, inflammaging has been proposed as a key mechanism in aging [15,17,22]. Inflammaging refers to the chronic, low-grade inflammation that typically accompanies aging, contributing to the pathogenesis of various age-related diseases [15,17]. This mechanism could explain the underlying the low-grade chronic inflammatory state seen in “natural” aging and multiple chronic conditions. Inflammatory biomarkers, such as C-reactive protein (CRP), interleukins 1 and 6 (IL-1 and IL-6), and tumor necrosis factor-alpha (TNF-α) have all been reported to be increased in the circulation of the elderly and patients with chronic lung disease [23,24,25]. Both natural aging and accelerated aging in chronic lung disease are states of chronic systemic inflammation, which affect almost every bodily system resulting in the development of multiple chronic conditions [26,27,28].

### 2.1. Aging and Changes in Body Composition and Metabolism

The changes in body composition in chronic lung disease are similar to those reported in natural aging [29,30]. The changes in body composition that occur with aging have a systemic dimension with a direct relationship between increased body mass index (BMI), fat mass, and CV risk factors, independent of age and ethnicity [29]. In natural aging, structural and functional musculoskeletal changes contribute significantly to an increased risk of sarcopenia, and, in parallel with an accumulation of fat and a loss of bone mineral density, lead to osteopenia and osteoporosis [29,30,31]. Such changes associated with natural aging are observed in COPD, but in an accelerated fashion [32]. Altered body compositions are an important factor in a reduction of physical activity with aging, although reduced physical activity itself can cause some of these changes [29,30,31,32,33]. Loss of muscle mass and function due to changes in muscle fiber structure and biochemistry were recognized early on as factors in the loss of functional capacity, while, more recently, increased fat mass and obesity leading to a reduction in the muscle-to-fat mass ratio have been shown to be important determinants of the level of physical activity [29,30]. This interaction is complex, but it is likely that maintained physical activity reduces the development of disability through a reduction in fat mass as well as the maintenance of skeletal muscle function [29,30].

Obesity, as a result of changes in body composition and physical inactivity, is linked to hypertension in addition to dyslipidemia and both are linked to T2D [34,35,36,37]. A potential mechanistic factor is the production of a change in the balance between the pro-inflammatory and anti-inflammatory mediators produced by adipose tissue, such as leptin (pro-inflammatory) and adiponectin (anti-inflammatory), which may contribute to altered skeletal muscle metabolism and function [37]. The combined impact of altered body composition and metabolic abnormalities subsequently leads to endothelial and vascular dysfunction, which is linked to accelerated atherosclerosis [35]. Although the presence of these changes appears to increase with age, lifestyle changes may also influence the progression of these physiological changes [36].

### 2.2. Aging and the Cardiovascular System

Aging is associated with many changes in cardiovascular structure and function, which makes it the main risk factor for CVD [10]. The prevalence of CVD rises sharply over the age 65, accounting for 40% of deaths of individuals between the ages of 65 and 74 and almost 60% of over 85 year olds, with 80% of all CVD-linked deaths occurring in individuals over 65 years [29,38]. This relationship occurs irrespective of sex, with the prevalence of CVD increasing from 40% in men and 34% in women, aged 40–59 years, to 70 and 71% in men and women aged 60–79 years, respectively [39]. The Framingham Study demonstrated that changes in behavioral and socioeconomic factors with aging result in the accumulation of risk factors, such as obesity and central arterial stiffness, that contribute to the development of CVD [40].

An important underlying factor causing changes in cardiovascular function and increasing cardiovascular risk with age is the development of atherosclerosis, a progressive inflammatory process affecting the endothelium and other layers of the arterial wall [41]. Atherosclerosis is considered to be due to cumulative damage initiated through a variety of insults, including endothelial dysfunction, the proliferation of vascular smooth muscle, and the deposition of extracellular matrix proteins [41,42,43]. The injury mechanisms are localized with external oxidative stress, inflammation, and changes in gene expression in the cardiovascular system, which are all processes that influence cardiovascular aging [43,44,45,46].

In patients with chronic lung disease, the most frequently encountered morbidity is CVD and this is associated with a two-to-threefold increase in the risk of cardiovascular events including death, even after an adjustment for smoking, hypertension, hypercholesterolemia, and obesity [47]. The association between CVD and chronic lung disease is now well established and accounts for upwards of 30% of deaths [48,49]. The link between CVD and chronic lung disease may be attributed to a number of shared factor risks such as smoking, inhaled particulates, inflammation, endothelial dysfunction, and protease/antiprotease imbalance [50,51]. These factors may lead to premature vascular aging and subclinical atherosclerosis, which impact the heart through structural and functional changes in the left ventricle (LV) [36,51].

### 2.3. Aging and the Respiratory System

Similar to the cardiovascular changes associated with aging, the respiratory system undergoes several changes with aging including a decline in pulmonary function [52,53]. These non-pathological changes resemble those seen in chronic lung disease and are important because they independently affect morbidity and mortality in the older population [54]. With aging, there are gradual structural changes in the thoracic cage and its musculature, including sarcopenia, causing increasing degrees of kyphosis, which alters the relationship between the ribs and intercostal muscles causing less efficient respiratory function, with a progressive loss of respiratory reserve [53,54,55]. Pulmonary function changes include a leftward and upward shift in the pressure–volume curves due to loss of elastic recoil of the lung leading to age-related hyperinflation [55,56]. Such changes are associated with a decline in the forced expiratory volume in one second (FEV_1_) and forced vital capacity (FVC), with the rate of annual decline with age being greater for the FEV_1_ after the age of 65 [56,57,58].

Changes in lung defenses occur with reduced mucociliary clearance of particulate matter and the impact of reduced respiratory muscle force reduces cough clearance with age [59,60]. The lungs may be exposed to both local pulmonary and systemic oxidative stress due to the inhalation of exogenous oxidants in air pollution and cigarette smoke, while local inflammatory leukocytes produce these endogenously either in response to inhaled material, such as P2.5 and P10 particulates, or to infection, leading to local tissue injury [61]. Such exposures and the host inflammatory response may be factors in the aging of the respiratory system. Possibly of greater importance is the reduction in the quality of the innate and acquired host responses [59,60,62]. A background activation of the innate leads to inflammaging and associated immunocellular aging, which leaves the host at risk of a poorer response to tissue injury or infection [30,31]. Changes in acquired immunity also occur with less naive T lymphocytes with aging, which impairs the host response to new infections as the majority of lymphocytes are memory cells [63].

### 2.4. Aging and the Central Nervous System

Individuals experience changes in the central nervous system as a normal process of aging [64]. The most common neurodegenerative change associated with age is cognitive impairment [65]. A number of brain regions, including the prefrontal, frontal, and limbic, exhibit hypoperfusion, ischemia, and atrophy [64,65]. Nevertheless, the neurodegenerative changes in the central nervous system appear precipitately in chronic lung disease (i.e., COPD) as a part of extrapulmonary manifestations [66,67]. A number of studies reported that patients with chronic lung disease suffer from cognitive impairment independent of age, sex, smoking status, and educational level [68,69,70,71]. The exact mechanism is not well understood, but it is likely to be multifactorial [65,71]. The proposed mechanisms include lung severity, increased circulating inflammatory biomarkers, and hypoxia-induced brain damage [72,73,74]. Increased lung severity in parallel with reduced oxygenated blood flow to the brain may cause neural damage leading to cognitive impairment [73,74]. The increased number of circulating inflammatory biomarkers contributes further to a reduction in execution function and delayed memory as a result of the grey matter atrophy of the frontal cortex and limbic system structures [73].

Neuroimaging studies showed structural and functional alterations in the brain of patients with chronic lung disease, and an abnormal activation of multiple brain regions [73,74]. In patients with COPD, the frontal and parietal lobes have been shown to have reduced blood perfusion and diffuse injuries of the grey and white matters in multiple brain regions, including the limbic and paralimbic systems [75,76].

## 3. Interlink Between Aging, Frailty, and the Lung

With aging, individuals are predisposed to the development of chronic diseases. The accumulation of two or more biological abnormalities (i.e., diseases) is termed multiple chronic conditions [1]. The major impact of multiple chronic conditions is the progressive impairment of physical performance and wellness, including cognitive and psychosocial wellbeing as captured in the assessment of frailty, and the state of disability. Frailty can be defined as a failure to respond to external stresses and maintain normal homeostasis [77]. In clinical gerontology, frailty and multiple chronic conditions are considered aging biomarkers for recognizing individuals at increased risk of disability, recurrent hospital admission, and mortality [3]. Frailty and multiple chronic conditions are interconnected, and having multiple chronic conditions is a major determinant of frailty [1,3]. Frailty can be described and assessed as a number of clinical changes indicating reduced physical function or as a multidimensional, risk state quantified by the number rather than the nature of deficits in physiological systems [27,28,77,78,79,80]. Using these concepts, frailty can be quantified, with both approaches giving similar insights into this state [79]. Determining deficit accumulation can be used to derive a frailty score based on the Comprehensive Geriatric Assessment (CGA) questionnaire. This score, expressed as a frailty index (FI), is used to determine health status and function while allowing for comparisons between different populations [81].

In the general population, frailty is associated non-linearly with increasing age, with the female gender, functional dependence, systemic inflammation, and chronic diseases [82]. Frailty better predicts adverse outcomes than chronological age independent of co-existing medical conditions and is associated with an increased risk of falls, hospitalization, residential care, reduced health related quality of life (HRQoL), progression to disability, and increased mortality [78,82,83]. In a female cohort, the risk of frailty increased with inflammatory comorbidities, e.g., the combination of pulmonary disease with anemia carried a risk ratio of 5.57 when compared with control subjects who had fewer morbidities. In this cohort, frailty was related non-linearly to the number of abnormal physiological systems, which was more predictive of frailty than the degree of abnormality in any one system, which resembles the impact of multiple chronic conditions in chronic disease [82]. The physiological deficits associated with frailty include sarcopenia and a progressive loss of physical capacity, a loss of bone mineral density, enhanced systemic inflammation, a reduction in HRQoL, and increasing cardiovascular morbidity and mortality [83]. The development of frailty is a consequence of the accumulation of system failures and the subsequent multiple chronic conditions. The similarities between the accumulation of deficits in aging and in chronic lung disease suggest that frailty is likely to be a common feature in such chronic lung disease.

A recent systematic review and meta-analysis found that the prevalence of frailty in individuals with chronic lung disease ranged from 9% to 28% according to Fried’s criteria [84,85,86]. Chronic lung disease increases the risk of frailty, and individuals diagnosed with chronic lung disease are at double the risk for frailty compared to non-lung disease individuals. The mechanism(s) which underlines the contribution of chronic lung disease to the development of frailty remains unknown. However, chronic lung disease and frailty share similar predisposing factors including smoking, systemic inflammation, physical inactivity, and lifestyle factors, including social deprivation [85,87]. In chronic lung disease, an individual’s body systems undergo remarkable changes such as loss of lung function, loss of muscle mass, and decreased physical activity mirroring similar changes which occur with aging and are features of frailty [28,33,35,73]. Individuals with the combined effects of chronic lung disease and frailty are at a greater risk of hospital admission for acute exacerbations and premature mortality [84,86]. Both chronic lung disease and frailty are progressive and associated with adverse outcomes. Nevertheless, frailty in chronic lung disease could be reversible by enrolling in a comprehensive pulmonary rehabilitation program that addresses the multiple chronic conditions associated with frailty and chronic lung disease [87,88]. Pulmonary rehabilitation especially involves prescriptions that consider symptom burden, as well as comorbidities that may enhance patient engagement and outcomes [87,88,89,90]. A recent report from the American Thoracic Society on Rehabilitation for People with Respiratory Disease and Frailty stated that pulmonary rehabilitation can address and reduce frailty in individuals with lung disease [91]. These findings collectively support the notion that frailty can be reversible in patients with chronic lung diseases through participation in comprehensive pulmonary rehabilitation programs that address the associated multimorbidities [87,88,89,90,91]. However, the long-term effect of pulmonary rehabilitation on frailty still needs to be explored in future research.

## 4. Treatment of Aging and Multimorbidity

The majority of available treatments are for established individual morbidities, though interest is growing in potentially preventative options. The established treatments can be considered pharmacotherapy and non-pharmacotherapy. Earlier recognition and treatment of the morbidities occurring in chronic lung disease, including hypertension and ischemic heart disease, together with preventative options, should reduce the predicted trends in cardiovascular morbidity and mortality [92].

### 4.1. Pharmacotherapy

Traditionally, multiple chronic conditions occurring as part of aging, such as ischemic heart disease, hypertension, heart failure, COPD, T2D, and osteoporosis are treated with accepted agents as individual diseases. Acceptance of the interrelationship between morbidities is seen in the use of cardio-protective options in T2D, with strict control of blood pressure, and the blood levels of lipids and glucose as recommended in various guidelines. Similarly, in chronic lung disease, clinical guidelines recommend the treatment of other morbidities as they would in non-respiratory settings [80]. This approach to chronic lung disease, in particular, using angiotensin-converting enzyme (ACE) inhibitors and β-adrenergic receptor antagonists, has been associated with reduced mortality and exacerbations in patients with chronic lung diseases such as COPD [93].

Statins are widely recognized for their lipid-lowering properties, but they also exhibit anti-inflammatory effects, notably reducing high-sensitivity C-reactive protein (hs-CRP) levels [94,95]. In the Jupiter study, 17,802 apparently healthy male and female participants with LDL cholesterol levels of less than 3.4 mmol/L and hs-CRP levels greater than 2.0 mg/L were randomized to either receive rosuvastatin, 20 mg per daily, or sham [94]. After a median follow up of nearly 2 years, the rosuvastatin group showed a reduction in hs-CRP levels by 37% compared to 17% in the sham group. Similarly, a network meta-analysis compared the effectiveness of various statins in patients with COPD [95]. The analysis indicated that fluvastatin and rosuvastatin had higher probabilities of reducing CRP levels compared to other statins, with fluvastatin showing the greatest potential. These findings suggest that statins may exert beneficial anti-inflammatory effects in chronic lung diseases by lowering hs-CRP levels, with variations observed among different statins. Further large-scale, randomized controlled trials are warranted to confirm these effects and to determine the optimal statin choice for managing inflammation in chronic lung diseases.

A new strategy to target accelerated aging for treating aging and chronic lung diseases is senotherapy [96,97,98,99,100,101,102]. Several novel targets for therapy have been identified, including PI3K-mTOR signaling, reduced anti-aging molecules, critical microRNAs, novel antioxidants, and the removal of senescent cells with senolytics by promoting apoptosis [103].

### 4.2. Non-Pharmacotherapy

Life-style changes including regular exercise and physical conditioning, smoking cessation, and dietary regulation are likely to impact on multiple chronic conditions in aging and in chronic diseases [104].

#### 4.2.1. Exercise and Physical Conditioning

Despite changes with aging, most older people can improve skeletal muscle force, as well as cardiovascular and respiratory function through sustained conditioning. Supervised resistance exercise appears to increase muscle strength and functional capacity in the elderly with sarcopenia, though there are few definitive studies [105,106]. Early initiation and the maintenance of functional capacity should reduce the predicted trends in cardiovascular morbidity and mortality among aged individuals [33,34]. Regular, moderate exercise produces multiple reductions in cardiovascular risk factors, including blood pressure, arterial stiffness, and lipid and glucose levels [1]. Similarly moderate to high levels of regular physical activity have been shown to have a preventative effect on the decline in airway function in active smokers and to reduce the risk of developing chronic lung disease [104].

#### 4.2.2. Calorie Control

There is increasing acceptance that dietary optimization, ideally combined with regular exercise, will reduce cardiovascular risk and might increase life span, while an increased fat mass and a sedentary lifestyle have the reverse effects [104,107]. Aging and chronic diseases are all associated with chronic systemic inflammation and oxidant stress, which may be related to calorie excess and low levels of exercise. Calorie restriction can protect against such effects and recent research suggests this effect may be mediated through longevity genes that maintain defenses against aging and age-related morbidities [108].

Calorie restriction increases life span in a number of species, though evidence for the benefits of long-term calorie reduction in humans is still evolving. Short- and long-term studies of a range of calorie restriction regimens demonstrate a reduction in the biomarkers of aging and cardiovascular risk [107,108,109]. Heilbronn et al. conducted a randomized controlled trial to explore the short-term effect of 6-month calorie restriction on various health biomarkers, including longevity, metabolic adaptation, and oxidative stress in overweight healthy individuals. In this study, the authors revealed that calorie restriction with or without exercise reduced body weight, fasting insulin levels, body temperature, resting metabolic rate, and DNA damage [108]. Furthermore, the results from the Comprehensive Assessment of Long-term Effects of Reducing Intake of Energy (CALERIE) of healthy individuals showed that the long-term effects of a 25% reduction in calorie intake over two years demonstrated a reduction in the inflammatory biomarkers and altered gene expression associated with various biological pathways, including metabolic adaptation, proteostasis, DNA repair, mitochondrial biogenesis, and inflammation [109,110,111,112]. Additionally, calorie restriction may reduce tissue fat deposits and fat cell size as well as increase insulin sensitivity, and it has been associated with a 40% reduction in carotid intima-medial thickness compared to controls on a standard American diet [113]. In other studies, using predefined multiple cardiovascular risk variables, the estimated 10-year risk was reduced by 30% with a 25% calorie restriction and 40% when this was combined with an exercise regimen [114].

A combination of calorie reduction or a more severe calorie restriction (10–30% reduction) for varying periods may have anti-aging effects [109,111,112]. This may be mediated by an enhancement of the health biomarkers associated with longevity such as improved insulin sensitivity, metabolic adaptation, and reduced oxidative stress. While these findings are promising, it is important to note that the degree of benefit from calorie restriction may vary depending on genetic factors and individual health conditions.

## 5. Expert Opinion and Practical Recommendations

Many chronic disorders are a component of a multimorbid state and mimic the accumulation of physiological deficits seen in natural aging. In terms of this parallelism, there is a growing body of evidence that chronic disease linked to multiple chronic conditions is a form of premature aging. The consideration of multiple chronic conditions in assessing chronic conditions adds a new dimension to the management of research directions in chronic disorders such as COPD and CVD. Thus, multiple chronic conditions either as a feature of aging or of chronic disease pose a number of important questions for healthcare systems, including how to deliver care to address multiple chronic conditions rather than responding to individual disorders. Within current healthcare systems, advances could be made by a simple modification of services. If cardiovascular clinics carried out spirometry, particularly on current and ex-smokers, and pulmonologists carried out regular blood pressure measurements and were prepared to follow through with simple cardiovascular investigations, and both measured glucose, they would likely be able to detect multiple chronic conditions and allow earlier intervention. This should be combined with glucose estimations and further investigation according to the accepted guidelines. Further development would be required to provide an integrated approach to multiple chronic conditions. There is a need for individual disease guidelines to reflect the likelihood that care needs to address multimorbidities and could be modelled based on those developed in gerontology.

## 6. Conclusions

Chronic lung disease and age-associated multiple chronic conditions share similar networks of pathophysiological mechanisms. Integrating multiple chronic conditions insights into clinical decision-making in patients with chronic lung disease will be essential for improving clinical, functional, and biopsychosocial outcomes. Early recognition of multiple chronic conditions patterns could improve risk stratification and guide the use of targeted interventions, including anti-inflammatory and cellular aging-modulating therapies. Furthermore, it may help identify the patients who would benefit most from integrated, multidisciplinary care models that include pulmonologists, geriatricians, cardiologists, physical therapists, and primary care providers. In practice, understanding a patient’s multiple chronic conditions profile could inform not only diagnostic and treatment strategies but also advanced care planning and quality of life considerations.

Future research should expand on multiple chronic conditions patterns with longitudinal, cellular, and systemic aging biomarkers studies to better understand the multifactorial nature of age-related chronic lung disease.

## Data Availability

No new data were created.
